# Triboluminescence dominated by crystallographic orientation

**DOI:** 10.1038/srep26324

**Published:** 2016-05-19

**Authors:** Kuifang Wang, Liran Ma, Xuefeng Xu, Shizhu Wen, Jianbin Luo

**Affiliations:** 1 State Key Laboratory of Tribology, Department of Mechanical Engineering, Tsinghua University, Beijing 100084, China; 2 School of Technology, Beijing Forestry University, Beijing 100083, China

## Abstract

Triboluminescence (TL) is an optical phenomenon that has a long and varied history with broad applications, such as damage detection, X-ray source, and mass health monitoring sensor. So far, the properties and mechanisms of TL remain not completely understood. The TL properties emitted during the sliding contact between Al_2_O_3_ and SiO_2_ surfaces were studied along different crystallographic orientations. In this study, the TL intensity of Al_2_O_3_ was significantly enhanced as Al_2_O_3_ surface was along a particular crystallographic orientation, which is an unconventional phenomenon. TL enhancement of Al_2_O_3_ was not affected by air atmosphere and atomic stocking mode of Al_2_O_3_. The enhancement mechanism of Al_2_O_3_ may be influenced by the surface state of Al_2_O_3_. This work provides a new method to control the intensity of TL and novel ideas to elucidate the TL mechanism.

Triboluminescence (TL) is a luminescence phenomenon by solid materials when they are stressed or fractured^1^,^2^. TL is also regarded as fracture, piezoelectric, and mechanical luminescence. Since the 20th century, this phenomenon has gained increasing attention because of its broad application^3^. TL has been successfully used in mass health monitoring sensor^4^, X-ray source, and damage detector^5^, among others. The triboluminescent properties of crystal materials are mostly investigated because many common crystal materials exhibit TL performance^6^.

TL properties of crystal are often affected by external factors, such as gas atmosphere, gas pressure, temperature, and speed. The photon emission intensity of Al_2_O_3_, ZrO_2_, and Si_3_N_4_ with a diamond stylus decreases with increasing number of carbon atoms in the hydrocarbon molecules^7^, whereas the emission intensity enhances to a maximum value at a particular n-butane gas pressure^8^. The TL intensities of NaCl and LiF doped with Br, Sr, Ca, and Pb decrease with temperature and disappear completely at 105 ± 5 and 180 ± 10 °C, respectilvely^9^. Hollerman *et al.*^10^ reported that the TL emission of Zn:Mn appears to be a function of speed for collision up to 6 Km/s. The doped impurities can change the TL properties of crystal materials; the TL emission intensity of ZnS:Mn is extremely strong, but TL properties are absent in ZnS^4^,^9^,^11^.

The physical properties of crystal structure also greatly influence TL. The discussion about TL properties of crystal with different space groups indicated that a non-centrosymmetric crystal structure is necessary but not sufficient for TL in crystal materials^12^,^13^. Hird^14^ reported that the intensity of TL emission during diamond polishing in ‘hard’ direction is greater than ‘soft’ direction. The crystallographic orientation of Al_2_O_3_ influences the atomic and electronic structures of alumina surfaces^15^. Brewer *et al*.^16^ investigated the fluorescence band at 3.0 eV, which was produced by photoexcitation in high-purity Al_2_O_3_ crystal. The results showed that the emitted light is plane polarized with the maximum intensity that occurs when the electric vector is perpendicular to the c axis of the crystal (E⊥c) and with the minimum intensity that occurs when the electric vector is parallel to the c axis (E ∥ c)^16^. Kurita *et al*.^17^ explored detailed atomic structures and electron states of stable and metastable surfaces of three important planes of Al_2_O_3_, namely, C plane [the (0001) surface], R plane [the (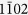) surface], and A plane [the (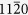) surface]. They found that the stoichiometric surfaces of the C plane have the lowest surface energy, followed by the stoichiometric surfaces of the R plane and then the A plane. In other studies, Cs-corrected high-resolution electron microscopy that combines first-principle calculations and image simulations was used to observe and investigate the quantitative and qualitative structures of (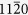) and (0001) surfaces^18^. Al_2_O_3_ possesses good TL properties, but is not clearly investigated. SiO_2_ is a common crystal material, and the investigation of SiO_2_ of TL properties is very less.

The TL properties of crystal materials in different crystallographic orientations are rarely reported. We explored the TL properties of Al_2_O_3_ along different crystallographic orientations by measuring TL emission during sliding with SiO_2_. We discovered an unconventional phenomenon that the TL intensity was enhanced several tens of times as Al_2_O_3_ plane was in a particular crystallographic orientation. This work may provide a novel method to control the intensity of TL.

## Results

### TL in ambient air

**Figure 1:**
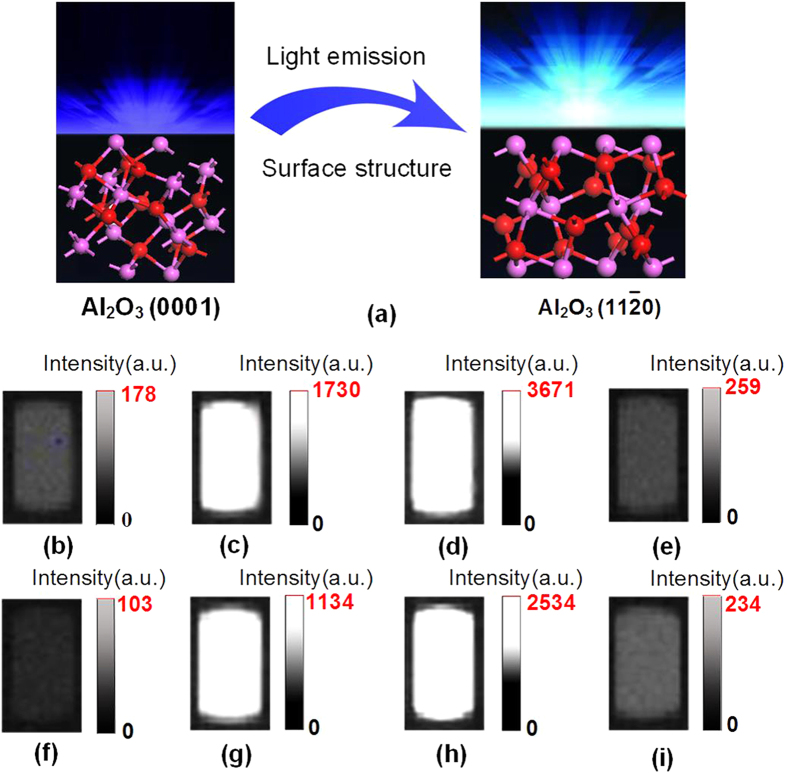
(**a**) Schematic of light emission of Al_2_O_3_ (0001) and (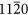) surfaces, and photon images emitted under F = 10 N and V = 33 mm/s during the sliding between SiO_2_ (110) and (**b**) Al_2_O_3_ (0001) with mean intensity I_(b)_ = 178, (**c**) Al_2_O_3_ (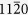) with I_(c)_ = 1730, (**d**) Al_2_O_3_ (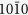) with I_(d)_ = 3671, and (**e**) Al_2_O_3_ (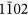) with I_(e)_ = 259, and during the sliding between SiO_2_ (003) and (**f**) Al_2_O_3_ (0001) surface with I_(f)_ = 103, (**g**) Al_2_O_3_ (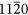) surface with I_(g)_ = 1134, (**h**) Al_2_O_3_ (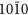) surface with I_(h)_ = 2534, and (**i**) Al_2_O_3_ (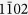) surface with I_(i)_ = 234.

Schematic diagram of light emission is shown in Fig. 1(a), the intensity of light emission of Al_2_O_3_ is greatly enhanced due to crystallographic orientation changed from (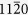) to (0001). The images of photon emitted in ambient air during the sliding contact between Al_2_O_3_ (0001), (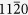), (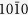), and (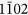) surfaces and SiO_2_ (110) under normal force (F) of 10 N and relatively shear velocity (V) of 33 mm/s are shown in Fig. 1(b–e). Images of (c) and (d) are much brighter than those of (b) and (e). The mean intensities of I_(b)_, I_(c)_, I_(d)_, and I_(e)_ were 249, 1605, 4512, and 222, respectively. The mean intensities of (b) and (e) are much weaker compared with those of (c) and (d). I_(c)_ is about 10 and 7 times higher than I_(b)_ and I_(e)_, and I_(d)_ is about 22 and 14 times higher than I_(b)_ and I_(e)_. The images of photons emitted during sliding between Al_2_O_3_ (0001), (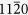), (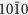), and (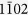) surfaces and SiO_2_ (003) are also shown in Fig. 1. In this condition, the intensity of TL has the same appearance although crystallographic orientation of SiO_2_ is changed. Thus, the TL intensities of Al_2_O_3_ (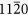) and (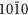) surfaces are significantly enhanced compared with the TL intensities of Al_2_O_3_ (0001) and (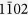) surfaces.

**Figure 2:**
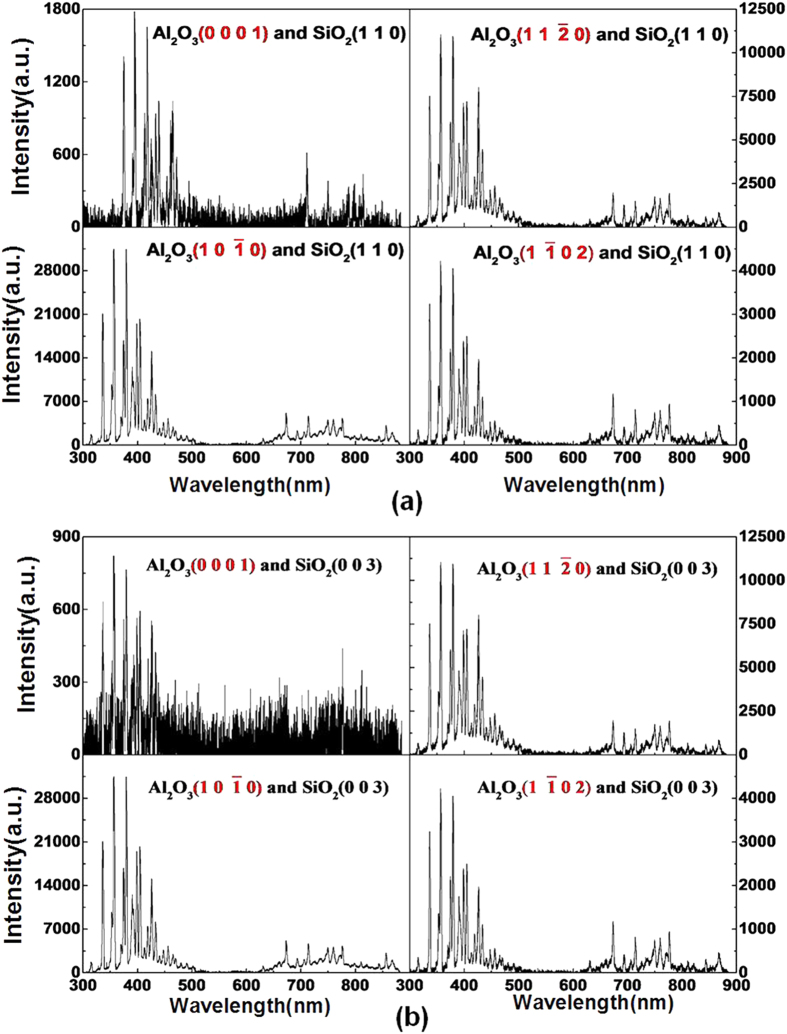
The spectra of photons emitted under F = 10 N and V = 33 mm/s in air: (**a**) during the sliding between (0001), (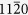), (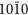), and (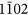) surfaces of Al_2_O_3_ and SiO_2_ (110), (**b**) during the sliding contact between (0001), (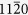), (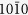), and (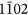) surfaces of Al_2_O_3_ and SiO_2_ (003).

The spectra of photons emitted in ambient air during the sliding contact between Al_2_O_3_ (0001), (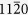), (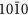), and (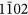) surfaces and SiO_2_ (110) under F = 10 N and V = 33 mm/s are shown in Fig. 2(a). Many sharp peaks in regions 300–450 nm and 600–900 nm are on spectra, but the spectra intensities of diverse Al_2_O_3_ surfaces are quite different. The maximum spectra intensities of Al_2_O_3_ (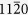) and (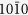) surfaces are much higher than those of Al_2_O_3_ (0001) and (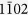) surfaces. The maximum spectrum intensity of Al_2_O_3_ (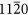) is nearly five times of spectra intensity of Al_2_O_3_ (0001) and (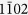) surfaces, in quantitative terms, while the maximum spectrum intensity of Al_2_O_3_ (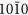) is more than 10 times than those of Al_2_O_3_ (0001) and (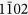) surfaces. As the crystallographic orientation of SiO_2_ is changed from (110) to (003), the spectra intensities of Al_2_O_3_ (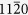) and (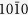) surfaces of Al_2_O are much stronger. Thus, the intensity of emission is enhanced, when Al_2_O_3_ (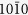) and (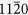) surfaces are sliding with SiO_2_.

**Figure 3:**
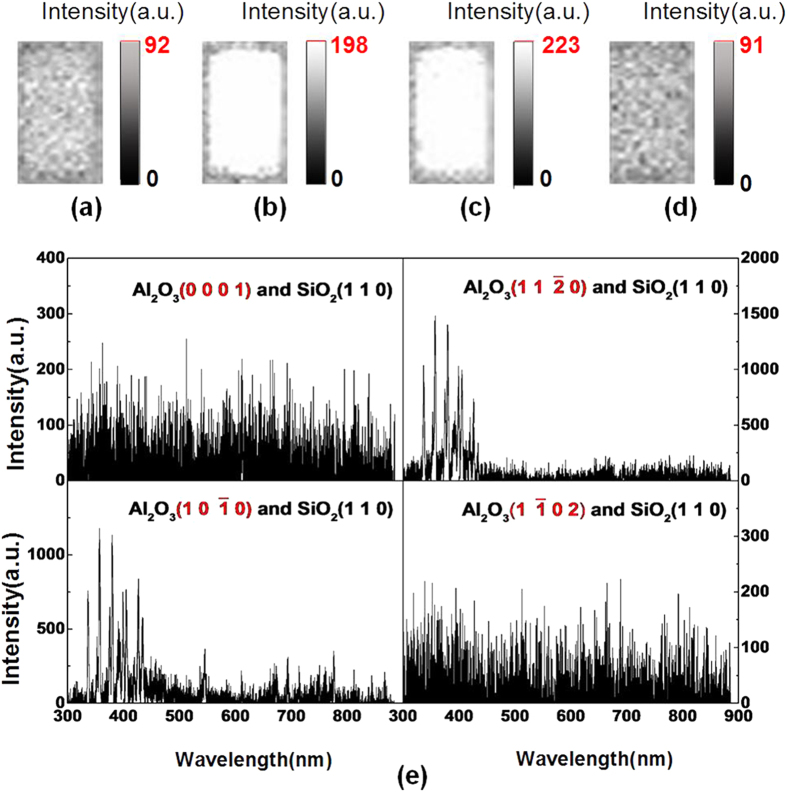
Photons under F = 10 N and V = 33 mm/s using a wire line in ambient air during the sliding between SiO_2_ (110) and Al_2_O_3_ (0001) (**a**) images with I = 92 and (**e**) spectra, Al_2_O_3_ (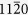) (**b**) images I = 198 and (**f**) spectra, Al_2_O_3_ (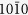) (**c**) images I = 223 and (**g**) spectra, Al_2_O_3_ (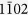) (**d**) I = 91 and (**h**) spectra.

We conducted other sliding experiments to study further the enhancement mechanism of TL intensity of Al_2_O_3_. The images and spectra of photons emitted in ambient air during the sliding contact between Al_2_O_3_ (0001), (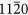), (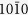), and (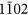) surfaces and SiO_2_ (110) surface under F = 10 N and V = 33 mm/s by using a wire line are shown in Fig. 3. The wire line that connects the holder and platform could reduce the external electric potential difference between Al_2_O_3_ and SiO_2_ to some extent. The mean intensities of images of Al_2_O_3_ (0001), (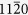), (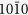), and (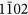) surfaces are 92, 198, 223, and 91, respectively. The mean intensities of images of Al_2_O_3_ (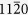) and (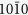) surfaces are higher than those of Al_2_O_3_ (0001) and (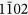) surfaces. The maximum intensity of images of Al_2_O_3_ (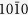) is nearly three times stronger than those of Al_2_O_3_ (0001) and (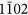) surfaces. In this condition, the photons emitted in ambient air are extremely weaker through eliminating the effect of external electric potential difference. The spectra of photons of Al_2_O_3_ (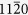) and (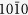) surfaces have sharp peaks as shown in Fig. 3(e), whereas those of Al_2_O_3_ (0001) and (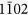) surfaces have no peaks. Thus, the TL intensities of Al_2_O_3_ (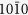) and (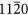) surfaces are much higher.

### TL in vacuum

**Figure 4:**
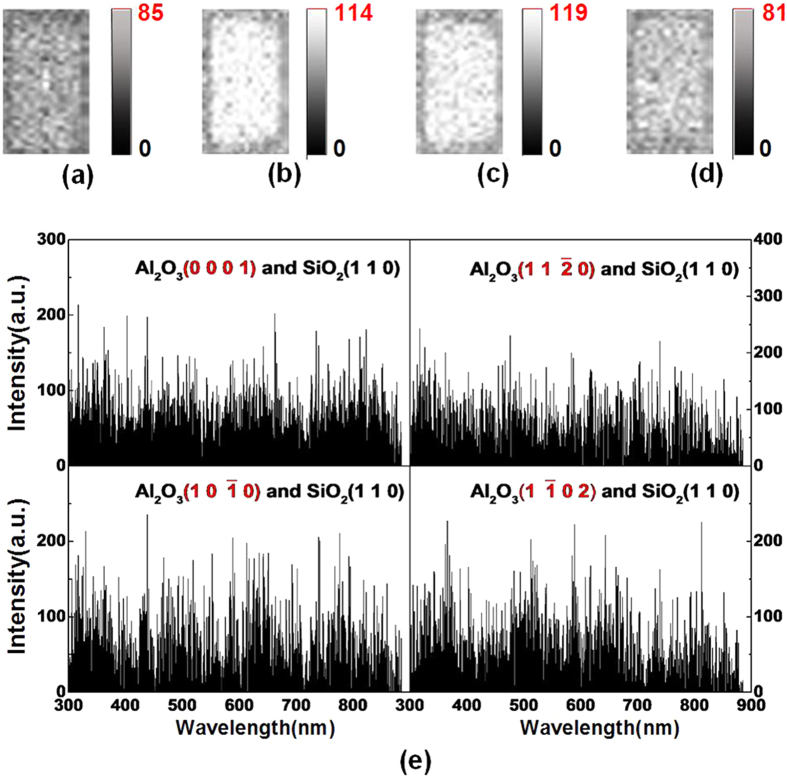
Photons under F = 10 N and V = 33 mm/s in vacuum during the sliding between SiO_2_ (110) and Al_2_O_3_ (0001) (**a**) images with I = 35 and (**e**) spectra, Al_2_O_3_ (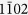) (**b**) images I = 114 and (**f**) spectra, Al_2_O_3_ (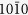) (**c**) images I = 119 and (**g**) spectra, Al_2_O_3_ (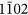) (**d**) I = 81 and (**h**) spectra.

To explore better the TL properties of Al_2_O_3_, we conducted subsequent experiments in vacuum instead of ambient air. Figure 4 shows the images and spectra of photons emitted during the sliding contact between Al_2_O_3_ (0001), (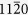), (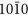), and (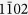) surfaces and SiO_2_ (110) in vacuum under F = 10 N and V = 33 mm/s. The spectra of Al_2_O_3_ in vacuum which are composed of instrument noise, have no characteristic peaks due to very lower photons intensity. The mean intensities of photon images of Al_2_O_3_ (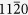) and (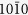) surfaces are higher than those of Al_2_O_3_ (0001) and (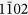) surfaces. The mean intensity of Al_2_O_3_ (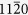) is nearly two times stronger than those of Al_2_O_3_ (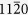) and (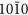) surfaces.

The spectra of photons emitted in vacuum have no peaks because of extremely low light intensity as shown in Fig. 4(e). In this condition, the TL properties of Al_2_O_3_ are also affected by crystallographic orientation, and the TL intensities of Al_2_O_3_ (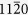) and (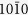) surfaces are enhanced.

### Friction and abrasion

The friction coefficients of the sliding contact between Al_2_O_3_ (0001), (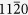), (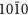), and (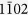) surfaces and SiO_2_ (110) are measured using a Universal Micro-Tribotester (UMT-3; Bruker, America), where the values of friction coefficients are 0.179, 0.258, 0.261, and 0.329. The friction coefficient of Al_2_O_3_ (0001) surface is lowest and that of Al_2_O_3_ (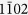) surface is highest. The friction coefficients of Al_2_O_3_ (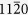) and (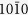) surfaces are much closer. The results prove that the TL intensity has no evident linear relationship with friction coefficient as the TL intensities of Al_2_O_3_ (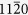) and (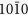) surfaces are much higher.

**Figure 5:**
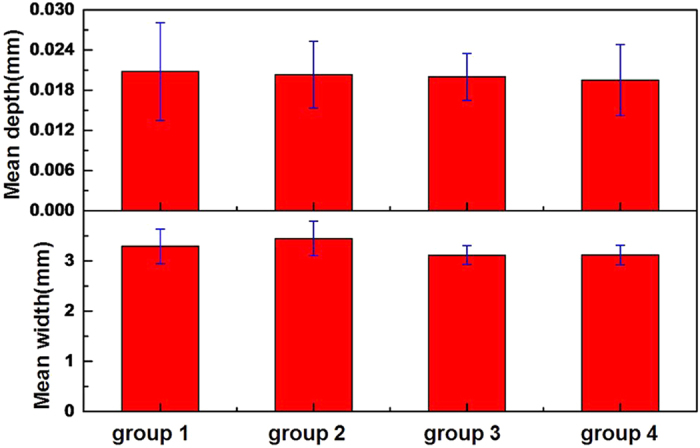
Mean depth and width of grinding crack of SiO_2_ (110). Group 1is SiO_2_ (110) sliding with Al_2_O_3_ (0001), group 2 is SiO_2_ (110) sliding with Al_2_O_3_ (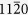), group 3 is SiO_2_ (110) sliding with Al_2_O_3_ (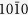), group 4 is SiO_2_ (110) sliding with Al_2_O_3_ (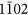).

The depth and width of grinding cracks on SiO_2_ (110) surfaces, which are sliding with Al_2_O_3_ (0001), (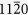), (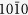), and (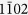) surfaces, are measured using a Talysurf (5P-120; Taylor Hobson, England). Figure 5 is the mean depth and width of the grinding crack of SiO_2_ (110) by sliding with Al_2_O_3_ (0001), (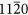), (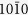), and (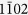) surfaces. The values of mean depth of four groups are close to 0.02 mm, whereas those of SiO_2_ (110) surfaces have no distinct differences. The results stated that abrasions of SiO_2_ (110) surfaces sliding with Al_2_O_3_ (0001), (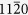), (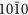), and (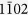) surfaces have no apparent variations. Thus, the TL intensity of Al_2_O_3_ is not influenced by wear of SiO_2_, and enhancement mechanism of TL properties of Al_2_O_3_ is dominated by crystallographic orientation.

## Discussion

**Figure 6:**
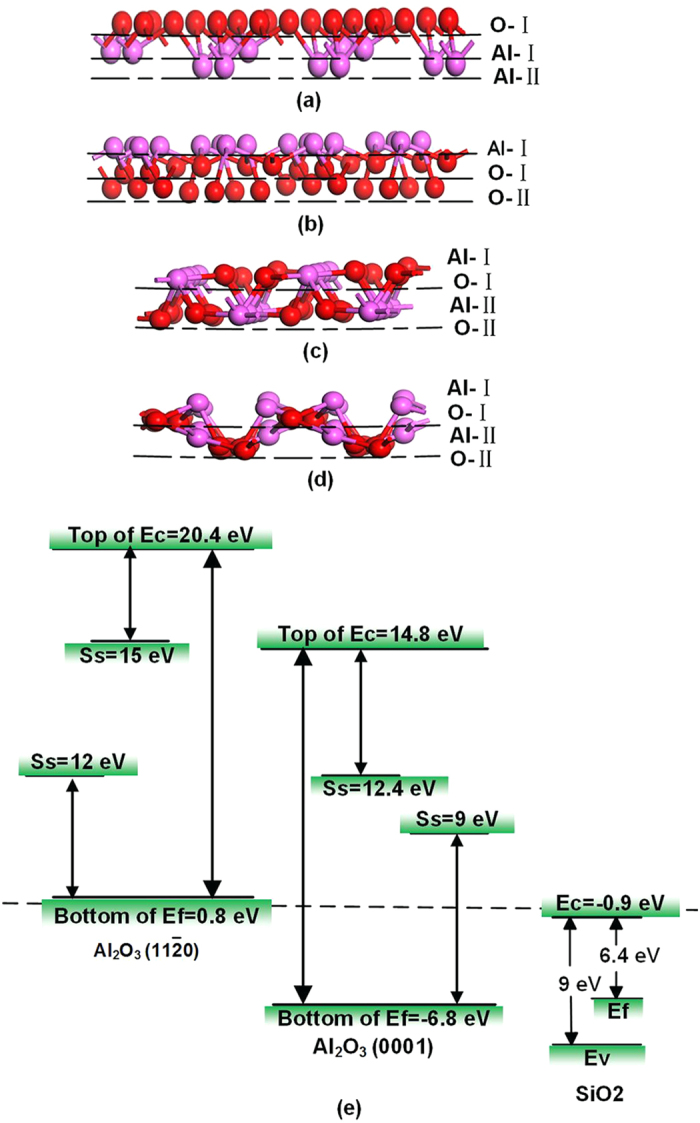
Side view of atom arrangement of Al_2_O_3_ surfaces: (**a**) (0001), (**b**) (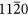), (**c**) (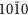), (**d**) (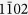); and (**e**): energy levels of Al_2_O_3_ and SiO_2._

The Al_2_O_3_ crystal is a hexagonal crystal, and the side views of atom arrangement of Al_2_O_3_ (0001), (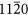), (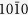), and (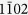) surfaces are shown in Fig. 6(a–d), respectively. In the hexagonal unit cell, the atoms are stacked along the [0001] direction in a sequence of an oxygen layer and Al double layers: -AlAlO_3_-AlAlO_3_-R (Fig. 6(a)). The atoms along the [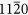] direction are a layer unit that consists of five atomic layers: an O layer constructed of one O atom in the 1 × 1 unit, an O layer constructed of two O atoms, an Al layer constructed of four Al atoms, and an O layer constructed of one O atom^17^. This -O-O_2_-Al_4_-O_2_-O- repeating layer unit has no dipole moment along the [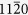] direction^17^. The atoms are stacked along the [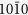] direction in a sequence of an O layer and an Al layer: -O-Al-O-Al-R- (Fig. 5(b))^18^. The 1 × 1 lateral unit of the (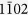) surface is a layer unit in a sequence of an Al layer constructed of two Al atoms and an O layer constructed of two O atoms. The -Al_2_-O_2_-Al_2_-O_2_- repeating layer unit has no dipole moment along the direction perpendicular to the (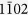) surface^18^. The atomic stacking mode of Al_2_O_3_ is extremely complicated along different crystallographic orientations, where (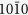) and (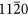) Al_2_O_3_ surfaces have no similar stacking mode. No regular discipline exists to indicate that the enhancement of TL intensities of (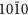) and (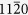) Al_2_O_3_ surfaces is related to atomic stacking mode.

The results of rough measurements of surface charge of Al_2_O_3_ and SiO_2_ by using faraday cup^19^ showed that Al_2_O_3_ surface was negatively charged and SiO_2_ was positively charged. Tribocharging mechanism is that electrons transferred from a surface with a low work function to a mating surface with a high work function^20^. In surface state theory, charge is exchanged between surface states in proportion to the difference between the effective or surface work functions of the two materials^21^. Surface potential difference is the fermi level difference between original surfaces as well as the work function difference^22^. In equation (1), V_C_ is the surface potential difference, *ϕ*_1_, *ϕ*_2_ are the work functions of two surfaces respectively^23^.


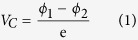


SiO_2_ surface has a lower work function than Al_2_O_3_ surface^24^,^25^. Thus, electrons are transferred from SiO_2_ to Al_2_O_3_, resulting in the former being positive and the latter being negative, then electric field between SiO_2_ and Al_2_O_3_ contacting surfaces is formed^26^. Electrons of ambient air molecules in electric field will be excited from ground level to the exited levels, then fall down to the lower or ground level, photons are emitted^26^. The sharp peaks of spectra are caused by the electrical breakdown of ambient gas^27^. The spectra peaks of photons in the region 300–450 nm are assigned to C^3^π → B^3^π electron transitions of N_2_^28^. Other sharp peaks of spectra are mainly due to the B^3^π → A^3^Σ electron transitions of N_2_ and the b^1^Σ^+^_g_ → X^3^Σ^−^_g_ electron transition in O_2_^27^. The pressure of vacuum chamber is between 1 to 10 Pa during the sliding experiment. Air molecules are much smaller under this condition. Thus, Photons emitted during sliding between SiO_2_ and Al_2_O_3_ in vacuum is very few, the spectra of Al_2_O_3_ in vacuum have no peaks.

As shown in Fig. 6(e), the band gap of SiO_2_ is 9 eV, and the conduction band (Ec) is −0.9 eV^29^,^30^. The surface states (S_S_) of Al_2_O_3_ (0001) and Al_2_O_3_ (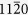) are 9 and 12 eV, respectively. The bottom of Fermi level of Al_2_O_3_ (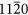) is 0.8 eV^31^,^32^. Al_2_O_3_ (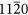) has two surface-state bands at 12 and 15 eV. The bottom of the Fermi level of Al_2_O_3_ (0001) is −6.8 eV, which is much lower than that of (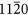). Al_2_O_3_ (0001) has two surface-state bands at 9 and 12.5 eV. The surface states of Al_2_O_3_ (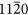) are much higher than those of Al_2_O_3_ (0001). Thus, enhancement mechanism of Al_2_O_3_ may be related to the energy levels of Al_2_O_3_, while much higher energy levels of Al_2_O_3_ (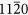) surface result in increasing TL emission.

The enhancement mechanism of TL properties of Al_2_O_3_ may be influenced by the surface state of Al_2_O_3_. The enhancement mechanisms of Al_2_O_3_ still need further exploration, and our work may provide a novel method to control the TL intensity.

## Methods

### Materials

**Figure 7:**
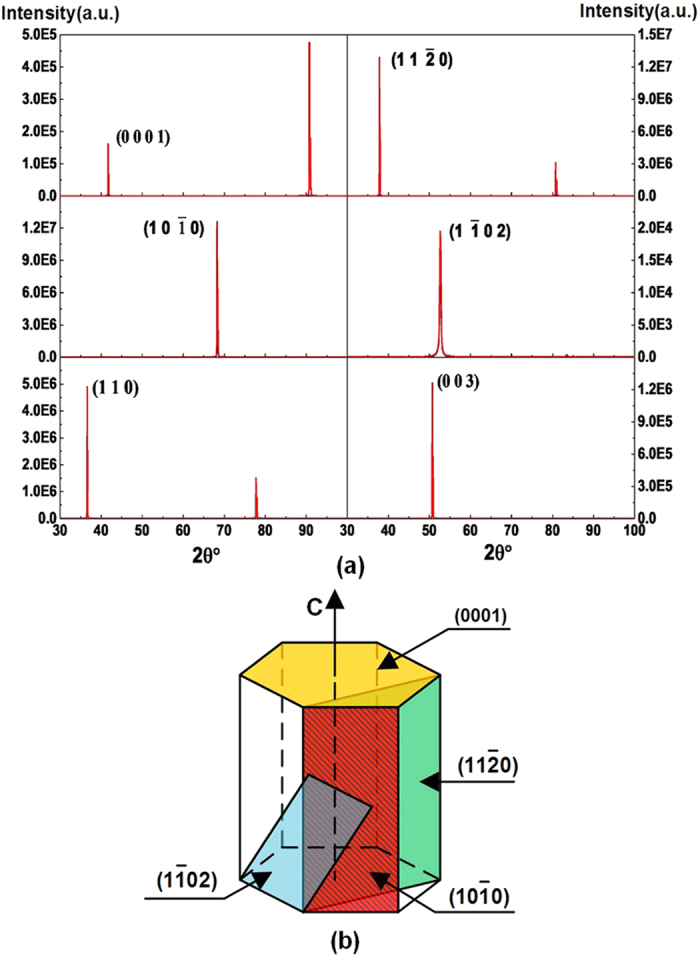
(**a**) Single-crystal X-ray diffractometer results of Al_2_O_3_ surfaces with miller indices (0001), (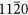), (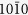), (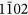), and SiO_2_ surfaces with miller indices (110) and (003). (**b**) Al_2_O_3_ planes in hexagonal unit cell.

SiO_2_ and Al_2_O_3_ crystals with trigonal and hexagonal crystal structures, respectively, were used in the sliding experiment. The two types of crystal planes of SiO_2_ were (110) surface by X cut and (003) surface by Z cut. The results of single-crystal X-ray diffractometer of SiO_2_ surfaces are shown in Fig. 7(a). Four surface planes of Al_2_O_3_ crystal, including C plane (0001), A plane (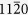), M plane (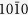), and R plane (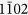)^33^, were used as shown in Fig. 7(b). (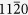) and (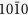) planes are parallel to axis, and (0001) plane is perpendicular to axis, (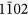) plane is crossed with C axis. The dielectric constant of sapphire at 298 K in 10^3^–10^9^ Hz interval is ∥C = 11.5, ⊥C = 9.3^34^. Dielectric constant of four planes of Al_2_O_3_ have little difference. The SiO_2_ with width of 3 mm and thickness of 2 mm and the Al_2_O_3_ with diameter of 30 mm and thickness of 2 mm were purchased from Shanghai Daheng Optics & Fine Mechanics Co. Ltd. The Vickers hardness of (110) and (003) surfaces of SiO_2_ are 1257 and 1167, respectively, and those of Al_2_O_3_ (0001), (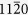), (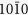), and (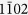) surfaces are 2060, 2119, 2076, and 2329, respectively. The roughness of (110) and (003) surfaces of SiO_2_ are 1.6 and 1.44 nm, respectively, and those of Al_2_O_3_ (0001), (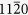), (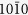), and (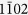) surfaces are 5.43, 5.21, 5.18, and 5.51 nm. In our experiments, Al_2_O_3_ (0001), (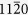), (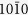), and (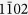) surfaces would be slid with SiO_2_ (110) and (003) surfaces, respectively.

### Experimental setup

**Figure 8:**
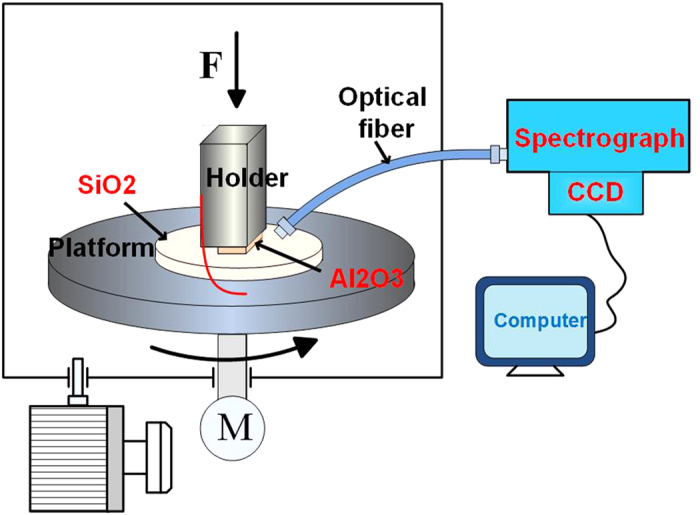
Schematic diagram of the sliding experiment setup for observation of the images and spectra of photons.

The schematic of the experiment setup used to observe the images and spectra of photons during sliding between SiO_2_ and Al_2_O_3_ is shown in Fig. 8. Optical fiber was used to gather light and then transmitted the light to a spectrograph (SP2500; Princeton Instruments, America). The images and spectra of photons were obtained with the spectrograph and CCD. The spectra of photons ranged from 300 nm to 900 nm, and the image of photons reflected the overall intensity of light.

### Experimental condition

SiO_2_ was adhered to the rotating platform along with the motor, and the Al_2_O_3_ wafer was fixed on a holder under a normal force of 10 N, as shown in Fig. 8. The bottom surface of Al_2_O_3_ wafer was sliding over the top surface of SiO_2_ wafer. The integration time (T) of CCD camera was 10 min, and the relative shear velocity (V) between Al_2_O_3_ and SiO_2_ wafer was 33 mm/s. The sliding experiment was performed in ambient air and vacuum. The vacuum pressure was between 1 and 10 Pa, and air humidity was nearly 10%. The red line in Fig. 8 is a removable wire line. The wire line connected the holder and the platform to reduce the influence of the external electrical potential difference. Each test was run three times, and mean was obtained to remove any discrepancies. The mean intensity of photon images is calculated by summing values of bright zone then dividing numbers of pixel points.

## Additional Information

**How to cite this article**: Wang, K. *et al.* Triboluminescence dominated by crystallographic orientation. *Sci. Rep.* **6**, 26324; doi: 10.1038/srep26324 (2016).
